# Coccidiostats and Poultry: A Comprehensive Review and Current Legislation

**DOI:** 10.3390/foods11182738

**Published:** 2022-09-07

**Authors:** Rui R. Martins, Liliana J. G. Silva, André M. P. T. Pereira, Alexandra Esteves, Sofia C. Duarte, Angelina Pena

**Affiliations:** 1 LAQV, REQUIMTE, Laboratory of Bromatology and Pharmacognosy, Faculty of Pharmacy, University of Coimbra, Polo III, Azinhaga de Sta Comba, 3000-548 Coimbra, Portugal; 2 Animal Research Centre (CECAV), University of Trás-os-Montes e Alto Douro, 5001-801 Vila Real, Portugal; 3 Centro de Investigação Vasco da Gama, Escola Universitária Vasco da Gama (EUVG), Av. José R. Sousa Fernandes 197, Campus Universitário de Lordemão, 3020-210 Coimbra, Portugal

**Keywords:** coccidiosis, coccidiostats, poultry, food contaminants, occurrence, legislation

## Abstract

Coccidiosis remains one of the major problems of the poultry industry. Caused by *Eimeria* species, Coccidiosis is a contagious parasitic disease affecting poultry with great economic significance. Currently, in order to prevent health problems caused by this disease, broiler farmers make extensive use of coccidiostats in poultry feed, maintaining animal health and, in some cases, enhancing feed conversion. The presence of unauthorized substances, residues of veterinary products and chemical contaminants in the food industry is of concern, since they may pose a risk to public health. As the use of coccidiostats has been increasing without any requirements for veterinary prescription, research and surveillance of coccidiostat residues in poultry meat is becoming imperative. This review presents an up-to-date comprehensive discussion of the state of the art regarding coccidiosis, the most used anticoccidials in poultry production, their mode of action, their prophylactic use, occurrence and the European Union (EU) applicable legislation.

## 1. Introduction

Over the past few decades, the poultry industry has significantly increased production, with great advances made in the areas of technology and genetics [1]. Concepts like clean, green and ethical, are the new challenges of poultry production systems [2]. Intensive production has led to an increase of stress and incidence of poultry diseases [3] due to the particularity that birds are raised together in large numbers and in high densities [4].

Of the numerous health problems affecting poultry, coccidiosis, caused by protozoa parasites, stand out as one of the most frequent, and their effects range from subclinical infections to the death of animals [5]. Coccidiosis is the most widespread and difficult to manage, especially in the broiler industry, causing intestinal lesions, poor weight income, poor feed conversion and reduced egg production. Moreover, it additionally favors further epidemic disorders, such as mycoplasmosis and colibacillosis [6], and, in its acute form, causes high mortality rates. The disease is not as vulgar when birds grow in extensive conditions [7].

In warm and humid environments, even when aseptic norms and good ranch operation are considered, coccidiosis is largely infectious and spreads from one animal to another by contact with infected feces, causing heavy influence on animal condition and well being, eventually leading to high mortality proportions [8]. When occurring with other diseases, this disease is more severe compared to its single occurrence, given its synergistic effect with other infections [9].

The economic consequences of coccidiosis are related to drops in animal production (improved feed conversion, growth decline and accelerated mortality), and to costs linked to treatment and forestallment. Worldwide, the annual financial impact of coccidiosis on commercial birds has been estimated at 2 billion euros [4,5]; costs can reach more than € 0.04/bird [10].

Originally, the disease was addressed as needed, when prompted by clinical complaints. However, in modern poultry production, the preventive control of coccidiosis is of paramount importance for facilitating rapid increases in the scale and intensity of poultry production, as well as to safeguard or improve zootechnical and financial results [11]. In Europe, current levels of poultry production would not be sustainable in the absence of an effective anticoccidial control programme. Therefore, almost all poultry granges have resorted to providing anticoccidial medicines as a feed additive to pullets and broiler breeders for 12 to 16 weeks, and to broiler chickens for nearly their entire lives. This practice greatly contributes to the protection of the health and welfare of animals, meeting the high European Union (EU) standards of safety and well being [12].

Coccidiostats are chemical products obtained by synthesis or produced by microorganisms which inhibit or destroy protozoa, the parasites responsible for coccidiosis [13]. Since 1940, under Directive 70/524/EEC of 23 November [14], they are employed in different food-producing animals, to prevent, inhibit and control parasitic protozoa of the genus *Eimeria* (the most predominant), *Isospora, Neospora* and *Cryptosporidium* belonging to the phylum Apicomplexa [5], appertained to as coccidia, causing a veritably infectious disease of the gastrointestinal tract in numerous tended animals [4]. They are most extensively employed as food complements in intensively bred species, such as pigs and birds, to maintain animal health and, in some cases, improve feed conversion [15]. According to the Regulation (EC) No 1831/2003 [13] of the European Parliament and of the Council of 22 September 2003, on additives for use in animal nutrition, coccidiostats are distinct from antibiotics used as growth promoters, whose primary action is on the intestinal microflora.

In the EU, eleven coccidiostats are authorized as poultry feed additives and divided into polyether ionophores (lasalocid (LAS), monensin (MON), maduramicin (MAD), narasin (NAR), salinomycin (SAL), semduramicin (SEM)) produced by different bacteria, and those of synthetic origin (decoquinate (DEC), diclazuril (DIC), halofuginone (HFG), nicarbazin (NIC), robenidine (ROB)), covering structurally diverse substances [16].

The means used for the forestalment and care of coccidian infections are nominated as anticoccidial drugs; those who kill the coccidial population are termed as coccidiocidal and those who prevent the replication and development of coccidial population are known as coccidiostats [17]. To protect against reinfection due to the omnipresence of the stadium oocystic disease, these drugs are administered from the first day of life of the birds up to seven days before slaughter [18]. This review presents an up-to-date comprehensive discussion of the state of the art regarding coccidiosis, the most used anticoccidial agents in poultry production, their mode of action, their prophylactic use, occurrence and the European Union (EU) applicable legislation.

## 2. Methodology

The available scientific literature was searched on Thomson Reuters ISI Web of Knowledge, PubMed, Science Direct, and Google Scholar databases. Combinations of at least two of the following keywords were used: “coccidiostats”; “Poultry”; “systematic review”; “residues”; “coccidiostat replacement”; “new approaches”; “carry-over”; “dietary exposure”; “validation”; “LC-MS/MS”.

The inclusion criteria comprised study design (cross-sectional and longitudinal observational studies), sampling procedure (random), timeframe (published between 1970 and 2020), legislation and language (English). Overall, the literature search included a total of 78 references published between 2002 and 2021.

## 3. Coccidiosis and Eimeria

In poultry, the *Eimeria* seven-day life cycle takes place outside (sporogony) and inside the host, in which both asexual and sexual (schizogony and gametogony) stages of reproduction ultimately occur [19]. Outside the host, in the environment, fresh oocysts (capsules with a thick wall protecting the parasite eggs), shed in faces as an undifferentiated stage (unsporulated), are not infective until they have sporulated [20]. This is a process that requires warmth and oxygen, and takes 24 to 48 h, for most poultry *Eimeria* species, depending on the environmental conditions observed [21]. Every sporulated oocyst contains four sporocysts, and each of those contain two sporozoites. It is a direct cycle that begins when active oocysts are orally ingested by the bird, in the infective transmission stage. In the digestive tract, after passing through the oesophagus, the oocyst undergoes mechanical action from the gizzard, releasing the sporocysts to the anterior part of the intestine, and into the duodenum by the action of pancreatic secretions (chymotrypsin and bile salts). Sporozoites (infective parasites) are than released in the intestinal lumen [22].

In the absence of treatment, these eukaryote host-specific, unicellular protozoa invade the intestinal tract of the host animal, where they multiply exponentially, causing damage to the intestinal cells, making it difficult to absorb nutrients, thus causing the development of diarrhoea and even bleeding [23]. Even in cases of slight infection, intestinal lesions caused by the reproduction of the parasite in the epithelial cells often facilitate other infections that can worsen the animal’s health status [24].

In chickens, at least seven *Eimeria* species are known to parasitize different portions of the intestine. According to Table 1, these have a life cycle that varies from four to seven days [5].

Eimeria species vary in pathogenesis, but *Eimeria tenella*, *Eimeria necatrix* and *Eimeria brunetti* are more pathogenic in chicken, giving rise to significant outbreaks of disease. When referring to broiler chicken, one must consider three species of economic importance: *Eimeria acervulina*, *Eimeria maxima* and *Eimeria tenella* [22].

This is typically a disease of young animals, since, after a first exposure to the agent, immunity develops quickly, protecting the bird against future infections. Unfortunately, there is no cross immunity between *Eimeria* species in birds, and subsequent outbreaks may occur for different species [25].

## 4. Anticoccidial Drugs

In 1939, for the first time, Levine cured coccidiosis in chickens using sulphanilamide. In the 1940s, by the widespread introduction of sulfaquinoxaline and recognition that this compound could be blended in feed during the poultry production, the infection could be averted [20]. This drug was used until 1948, when producers began to preventively use sulfaquinoxaline in feed. Nitrofurazone and 3-notroroxarsone were introduced in the 1950s, whereas in the early 1960s, amprolium and nicarbazin started to be used in broiler production [26]. In the 1970s, other highly efficacious synthetic drugs were introduced due to the emergence of resistance to the above chemicals. In 1971, significant progress in the control of coccidiosis was achieved with the introduction of monensin as the first ionophore to be used for this purpose. Previously, outbreaks of coccidiosis were frequent and more difficult to treat or prevent, since only synthetic coccidiostats were available [27]. It was considered extremely effective against all species of *Eimeria* infecting birds. Several other ionophores that have a similar mode of action, including narasin, lasalocid, salinomycin and semduramycin were discovered during the following years [5].

Currently, there are eleven different coccidiostats approved for use in the EU, including ionophores and synthetic compounds, in order to prevent the disease from spreading, to minimize parasite development and to improve immunity, simultaneously [24].

By interfering with the ability of ions to flow across cell membranes, disrupting the osmotic balance, ionophores cause parasite death. Currently, they are the mainstay of coccidiosis control. Synthetic compounds affect parasite metabolism, inhibiting parasite biochemical pathways [28,29].

### 4.1. Synthetic Compounds

Synthetic compounds are halofuginone (HFG), robenidine (ROB), diclazuril (DIC), decoquinate (DEC), nicarbazin (NIC), toltrazuril (TOL), clopidol (CLO ), Nequinate (NEQ), ethopabate (ETH), amprolium (AMP), sulfadimethoxine (SDM), sulfaquinoxaline (SQ) [30]. Each synthetic anticoccidial works against coccidia in a unique way. As a result of chemical synthesis, compounds of this type are often referred to as “chemicals”, and act by interfering with one or more stages of the life cycle of the parasite. As soon as the parasite invades the host´s intestine, they destroy its intracellular stages [31] These work well for severe infections, but in the long run, can result in increased resistance [4].

### 4.2. Polyether Antibiotics or Ionophores

Regarding authorized ionophores in the EU (monensin, salinomycin, lasalocid, narasin, maduramicin, semduramicin), whose name is derived from the Greek “ion phoros”, meaning “ion carrier”, the majority of coccidiostats used in Europe are obtained by the fermentation of *Streptomyces* spp. or *Actinomadura* spp. [32]. Ionophores transport ions (such as sodium (Na^+^), potassium (K^+^) and hydrogen (H^+^)) across hydrophobic membranes of the parasite wall. This increases the concentration of these ions inside the parasite, eventually resulting in the uptake of water through osmosis, causing the osmotic imbalance of the *Eimeria*, with greater loss of energy in the Na-K pump, causing the parasite vacuolization with the consequent swell and burst. There are some mithochondrial functions that are inhibited by an increased concentration of Na^+^ ions, such as its ability to oxidize substrates and hydrolyze ATP. The exchange of intracellular Na^+^ for extracellular Ca^++^ increment the concentration of Ca^++^ within the cells and cause cytotoxicity [31]. They usually act in the initial phase of life of *Eimeria* and can be coccidiostatic (interrupting the parasite cycle without destroying it) and/or coccidicides (killing parasites). Their mode of action makes them unsuitable for use as curative products. Ionophores are only able to destroy the parasite during the motile stages of the life cycle (sporozoites and merozoites), because they do not enter the intestinal cells. They effectively prevent coccidiosis, and at the same time, allow the development of natural immunity [5]. Their use in subtherapeutic doses contributes to weight gain in chickens [30]. Some bacteria, such as Clostridium perfringens, which causes necrotic enteritis, are also inhibited or killed by ionophore coccidiostats [24].

In some cases, mixtures are used consisting of a synthetic and an ionophore (nicarbazine/narasin) compound or two synthetic compounds (methylcyclindol/methylbenzoquate) [4].

### 4.3. Coccidiostats Programmes

Three types of drug programmes are used by the broiler industry: single drug programmes, “shuttle” drug programmes, and rotation programmes.

Single drug programmes involve the use of the same drug in the feed of a single flock. The prolonged use of this kind of drug programme will result in a gradual decline in efficacy because of the selection of resistant strains [5].

In “Shuttle” drug programmes, initially one type of synthetic drug is incorporated into the starter feed, then another type in the growers, then a third type in the finisher diet, and finally, a fourth type during withdrawal [33]. In this program, any resistance that may develop to one drug will be eliminated by another drug, and vice versa [34].

Rotation programmes involve the alternation of the use of two or more drugs at intervals of several months, in successive flocks. The majority of the rotation programmes involve the alternation of a synthetic drug employed in the starter and/or grower feed [34]. This will preserve their efficacy and help reduce the incidence of any strains resistant to ionophores.

## 5. Vaccines

The primary infection of avian coccidia can stimulate strong immune responses in the host by B and T lymphocytes of the gut-associated lymphoid tissues [35]. In the effort to control coccidiosis, vaccines are a great alternative to drugs [36]. To be effective and to ensure birds’ protection, vaccination must administered correctly [37], with vaccines containing sporulated oocysts of the most pathogenic Eimeria species (*E. acervulina, E. maxima, E. tenella, E. necatrix,* and *E. brunetti*). *E. maxima* stimulates the highest immune response in the host; even a single sporocyst can induce complete protective immune response, and five *E. maxima* oocysts can induce complete protective immunity [38].

Vaccines represent a natural control of coccidiosis as they induce an active immunity in 3–4 weeks, influenced by genotype of the host, history of infection and the amount of parasites [38].

The most recent advance is “*in ovo*” inoculation into 18-day embryonated eggs. This process consists of the deposit of the vaccine into the amniotic cavity of the embryo. As a result of this technique, vaccines can be delivered with precision and repeatability [39]. Present-day vaccination strategies give no confidence of same exposure to coccidia across the herd because it is difficult to administer the vaccine perfectly using these methods [20].

Some circumscriptions, like poor sanitary operation or inappropriate application of the vaccine, may lead to inferior performance compared to prophylactically treated birds [40].

However, Hamid et al. [11] note that vaccination combined with in-feed ionophores produced the best results in terms of commercial broiler productivity and immunity. The vaccines can be administered orally (feed in an edible gel or drinking water), via eye spray or via spray in the hatchery that facilitated their use as broiler chickens. However, its high cost (labour intensive and expensive) has hampered the widespread implementation of vaccination [41].

It has been more than 70 years since the first live coccidiosis vaccine was developed. Commercial vaccination of replacement breeders, laying pullets, and commercial broilers began in 1992 in the EU, followed in 2000 by a vaccine for commercial layers [27]. Presently, three types of vaccines are used: non-attenuated, attenuated and recombinant vaccines [42], comprising an assortment of non-attenuated and attenuated parasites of different species [40].

Non-attenuated vaccines have been widely used worldwide for about 50 years, and are effective in protection against the parasite [41]. They very often provide a solution when in-feed anticoccidials become ineffective.

The virulence of the attenuated anticoccidial vaccine is generally reduced due to the selection of earlier *Eimeria* isolates, that is, isolates that do not have a life cycle, compared to the *Eimeria* strain with a normal life cycle. Although vaccines attenuated anticoccidial drugs are still widely used today, the low degree of immune system protection requires that it be supplemented with adjuvants, which consist of different cytokines, to improve immunity [28,43]. To produce recombinant anticoccidial vaccines, the identification of specific surface and internal antigens in the *Eimeria* spp. at different stages of its life cycle is necessary, in order to induce an effective immune response [42].

The development of recombinant vaccines against field strains is limited by a lack of knowledge surrounding the antigens involved in potent immunity [42].

Coccidiosis control in poultry can be sustained over the long-term with rotation programs that alternate vaccination and drug use in successive flocks [27].

As DNA technology advances, vaccines that contain genes encoding Eimeria-derived protective immunogenic proteins will be developed to protect against coccidiosis [42].

## 6. Alternatives to the Use of Coccidiostats

In an effort to reduce the use of veterinary drugs in the food chain, alternative strategies have been sought to control coccidiosis.

Alternatives include natural products such as prebiotics and probiotics, plant extracts, fungal extracts and essential oils. 

In most cases, natural products do not directly target parasites, rather they stimulate the immune system and affect the intestinal microbiota [20].

By restoring the balance of the intestinal microbiota, prebiotics and probiotics help strengthen the immune system of birds.

Adding probiotics and prebiotics to animal feed helps restore the balance of the intestinal microbiota, eliminating pathogenic bacteria causing coccidiosis, such as Clostridium perfringens [31]. Probiotics combined with vaccination result in better performance than vaccination alone [42].

Phytotherapy is another alternative to coccidiostats, involving the use of extracts and essential oils from plants [44].

In recent studies, plant extracts and essential oils have been shown to effectively control coccidiosis. Namely, thyme and rosemary contain phytochemicals which aid in the prevention of coccidiosis. As a result of the pharmacological activities of their co-products, these plants are able to prevent and treat coccidiosis, which can be treated with feed additives by regulating *Eimeria* sp., host immunity, antioxidant status, and intestinal flora [45].

Using extracts of Moringa oleifera, or White Acacia, Abdel-Tawab, et al., 2020 found that the leaves have antioxidant, antiapoptotic and anticoccidial properties. E. papillata infected rats were shown to excrete 50.5% fewer oocysts in feces when treated with extracts of this plant, with an apparent reduction of parasitic stages within the cells, as well as an increase of the amount of goblet cells [46].

It is likely that the use of this plant will help control coccidiosis, as described by Ola-Fadunsin, et al., in 2013 [47].

All natural products must be evaluated for safety and toxicity before they are authorized for use, including plant and fungal extracts and essential oils [31].

## 7. Management and Control Approach

Controlling coccidiosis is very challenging due to the specific characteristics of the disease. Coccidiostat resistance contributes to this difficulty as well.

Before these medicines were available, handling was crucial to coccidiosis management. As long as immunity is not constituted, it aims to keep coccidia numbers at a minimum. In order to prevent overcrowding, breeders must ensure that birds have ample floor space, feeders and waterers [26].

Oocyst sporulation is dependent on humidity, and the greater the content, the more likely sporulation becomes. In commercial poultry houses, even those that are kept close to optimal sporulation temperatures, the temperature still remains high [39].

The amount of sporulated oocysts ingested and the severity of the infection are proportionally related to morbidity and host mortality. It is important to quarantine infected animals, as well as to remove dead animals’ bodies, to reduce the number of infectious organisms present in healthy animals. When an animal is infected, it should be possible to separate it from the others, preventing the transmission of the infection to healthy animals [48].

Good farm management is recommended, including the use of a quality ventilation system, litter hygiene around water drinkers, and the application of a fresh top layer before housing the chicks, thereby reducing sporulation of oocysts [33].

It is important to change the entire dress of caretakers before visiting a newly constructed poultry house to prevent the spread of resistant oocysts [49].

In order to combat coccidia, good hygiene practices and the removal of contaminated feces are the main goals of sanitation [26]. According to reports, only methyl bromide, carbon disulphide, ammonia, or phenols can kill oocysts [39]. Clinical coccidiosis can be prevented by using coccidiosis control programmes such as shuttle and rotation.

A number of recommendations have been made by the United States Department of Agriculture (USDA), including: increased vaccination of animals; improving the rations provided to the animals through diet adjustments and by adding appropriate trace elements, probiotics, and immunity boosters; and by increasing the nutritional quality of the rations;

Upon receipt of animals, tests must be conducted to ascertain their health status, so that only healthy animals are purchased [50].

## 8. Legislation

The substances and preparations used in the European Union (EU) to treat or prevent diseases in animals are normally regarded as veterinary medicinal products, and are regulated under the veterinary medicines and related legislation, particularly for their safety and efficacy. The exception of these rules are the coccidiostats group and their related formulations [51].

The European Food Safety Authority (EFSA) and the European Medicines Agency (EMA) are respectively responsible for the scientific evaluation of the safety of feed additives and veterinary medicinal products.

The European Commission monitors residues in meat during the commercial use of anticoccidials in order to ensure safety and effectiveness. For illustration, the Rapid Alert System for Food and Feed (RASSF) is a monitoring design where public authorities report data about deleterious products set up each day [52].

To illustrate, in Portugal, The National Residue Control Plan consists of a surveillance system that aims to analyse and highlight the risks of residues in foodstuffs of animal origin, and to clarify the reasons for the presence of such residues in food, making all players in the production chain responsible [53].

The National Residue Control Plan (PNCR) complies with the provisions of Decree-Law no. 148/99 of 4 May, and Decree-Law no. 185/05 of 4 November, and its amendments [53], and its elaboration and coordination of the competence of the General Directorate of Veterinary (DGV), current General Directorate of Food and Veterinary (DGAV)

The sampling is carried out by technicians from DGAV and the food and economic security authority (ASAE) with the following distribution: DGAV—in slaughterhouses, aquaculture, fairs and animal farms of the species that are intended for food production; ASAE—in slaughterhouses, cutting and hunting treatment establishments, in dairy farms, in apiaries and in egg classification centers [53].

The PNCR addresses three major groups of compounds: banned substances, veterinary drug residues and environmental contaminants.

To achieve food safety, it is critical to regard all angles of the food chain in its constancy, in a farm to fork strategy, as each component may have a potential impact on food safety, the unintentional or deliberate contamination of feed, its adulteration and fraudulent or other incorrect practices related to it, may affect food safety directly or indirectly [54].

### Maximum Residue Limits (MRLs) in Food and Acceptable Daily Intakes (ADIs)

Anticoccidial drugs improperly used in poultry have led to drug-resistant varieties, and the presence of residues in meat products that are inadmissible for human well being, due to their toxicity [55].

Therefore, in 1990, several countries made notification of coccidiostat residues in meat, eggs and other tissues. As a result, coccidiostat residues in food are currently being studied and monitored more closely [15].

The correct use of mandatory withdrawal periods could control the residue problem, however, under practical farming conditions, such obligations are not frequently upheld. Besides, due to the availability of so many coccidiostats, many farmers switch compounds to prevent drug resistance from building up over time. Consequently, most poultry are given feeds containing medicines for the greater part of their lives [56].

To achieve effective control of coccidiosis, a scrupulous biosecurity plan, in addition to vaccination and chemotherapy programs, is required [20].

To protect public health, maximum residue levels (MRLs) for the carry-over of anticoccidials in feed were established. A risk assessment conducted by the CONTAM Panel of the European Food Safety Authority (EFSA) revealed that a level equivalent to 5% of the maximum authorised concentration in feed would not pose a risk to consumers. Based on the species and categories of animals, MRLs were set at 1% or 3% of maximum authorised concentrations. A 1% level was established for animals during withdrawal periods, for animals producing food continuously (laying hens and dairy cattle) and for species particularly susceptible to the toxic effects of specific coccidiostats [57].

To cover and prevent undesirable contamination of animal products for human consumption, EU countries have established surveillance programs focused on ionophores and NIC in eggs and poultry meat, which are the main markers of the food residue promoting coccidiostats [30].

Despite the notification that the ingestion of pure molecules caused acute toxicity in humans, the primary concerns are the chronic toxicity related to long term exposition, which can cause hypersensitive reactions due to the unbalance of the dynamics in the gastrointestinal flora, and changing the microflora of the gut to develop microbial resistance to antibiotics [58].

In food products, Maximum Recommended Levels (MRL) are determined by calculations based on Acceptable Daily Intakes (ADIs). A residue’s ADI is an estimate (based on animal studies) of how much can be consumed repeatedly without causing health problems [59].

On the basis of toxicological data, the European Food Safety Authority (EFSA) and the Joint FAO/WHO Expert Committee on Food Additives (JECFA) determined acceptable daily intakes (ADIs) for all regulatory anticoccidials, with HFG (0.00003 mg/kg body weight/day) having the lowest (0.2 mg/kg body weight/day) values. As a result of toxicological data, the European Food Safety Authority (EFSA) and the Joint FAO/WHO Expert Committee on Food Additives (JECFA) have established acceptable daily intakes (ADIs) for all regulatory anticoccidials, ranging from the lowest value (0.00003 mg/kg body weight/day) for HFG to the highest value (0.2 mg/kg body weight/day) for NIC.

Food-producing animals and animal products are subject to European Union Regulation (EU) No 37/2010, which establishes maximum limits for residues of veterinary medicinal products. Commission Regulation (EC) 1881/2006 lays down the maximum limits for animal products containing certain contaminants [60].

For approved veterinary medicines and pesticides, EU and national regulations specify maximum residue limits (MRLs), and for contaminants in foods of animal origin, maximum levels (MLs) [61]. As shown in Table 2, MRLs were established in the EU for eleven coccidiostats [62].

The Commission Decision 97/747/EC also specifies the levels and frequency of sampling for certain animal products.

According to Article 31 of Regulation EC 178/2002, the European Commission (EC) asked the European Food Safety Authority (EFSA) to produce an annual compilation of the monitoring results obtained under the provision of Council Directive 96/23/EC.

## 9. Monitoring and Occurrence of Coccidiostats Residues in Food

Under Directive 96/23/EC, national authorities are required to develop and implement residue control plans, and key obligations are outlined for primary producers and processors. A residue control plan approved by the EU is also required for countries exporting to the EU. Table 3 presents the Production of poultry, number of target samples and the percentage of samples tested/200 t(a) from 2007–2019, whereas Table 4 exhibits the production volume and number of targeted samples collected for poultry, and Table 5 shows the different residues found in live animals and animal products in 2019.

According to the 2019 NRCP in the EU, 0.03% of chicken muscle samples were non-compliant and 0.21% in eggs. The compounds with the highest frequency of detection were halofuginone, lasalocid, monensin, and toltrazurilsulfon. Thus, it was found that in the EU in 2018, the most used compounds were ionophore coccidiostats. In the 2017 report, ionophore coccidiostats were also the most used, but the most frequently detected compound was salinomycin [63].

There has been a significant decrease of non-compliant samples for anticoccidials (B2b) in poultry since 2009. Coccidiostat levels in non-target feed have decreased due to sensitivities and measures following the implementation of the Commission Directive 2009/8/EC [63].

Adding new MRLs for nicarbazin in muscle, kidney and liver, respectively, at the end of 2010, led to a significant reduction in non-compliances in poultry meat. Before 2010, any tissue sample containing more than 200 grams of nicarbazin per kilogram was considered non-compliant. In light of the current MRLs for nicarbazin, most of these pre-2011 non-compliant results would now be considered compliant [15].

Table 6 presents data regarding occurrence obtained from different research studies. As shown, a study carried out in Poland between 2007 and 2010 analysed 3718 samples of chicken, turkey, eggs, water and feed for the control of coccidiostat residues and found that, as in the present study, ionophore coccidiostats were the most used in the prevention of coccidiosis. Of these, lasalocid was the most frequently detected (32.8% of the total non-compliant samples). In 2007 and 2010, these authors detected lasolacid, salinomycin, nicarbazin and maduremicin in the aforementioned samples. In 2008, in addition to the compounds detected previously, samples with robenidine were also detected [65].

On the other hand, in a study carried out in Italy between 2012 and 2017, which analysed 202 samples of beef, pork, sheep, rabbit and chicken muscle, with 82.2% of the analysed samples being chicken, the analysed samples revealed a frequency of contamination that varied between 14.7% and 48.7% during the period considered, and synthetic coccidiostats were the most detected. Ionophore compounds were detected in only six samples (2.8%), and of these, the coccidiostats detected were mainly lasalocid and narasin. Synthetic coccidiostats were detected in 66 samples (31.7%), of which the most detected were nicarbazin and diclazuril. In no meat sample was more than one coccidiostat detected and no sample exceeded the established MRLs [30].

In a study carried out in accordance with the PNRC of Greece [66] 82 chicken and egg samples were analysed, in which only one chicken sample exceeded the MRL. However, coccidiostat residues were detected in 25 samples below CCα. The compounds that showed the highest frequency were decoquinate, salinomycin and maturemycin. Similar to this study, some samples showed more than one coccidiostat, but the most frequent combination was decoquinate and salinomycin. In Spain, a study analysed chicken muscle, beef muscle and its derivatives: kidney and liver, eggs, milk and pork fat. The coccidiostats monensin and salinomycin exceeded the MRL value in pork fat, narasin in eggs and salinomycin in bovine kidney. The maximum concentration values detected in this study were 11.14 µg/kg for decoquinate, this value is lower than those observed in the aforementioned studies, especially when compared to the maximum concentrations of 2800µg/kg found in chicken muscle. Eggs were not considered as a matrix for this study, but they are important because they are a by-product of the poultry industry, thus indicating problems at the level of laying hens that receive rations with coccidiostats and do not respect the withdrawal period of coccidiostats, as defined by the legislation, putting consumer health at risk [67].

**Table 6:** Frequency (%) and levels (μg/kg) of coccidiostats in poultry meat reported in the scientific literature.

Country	Analysed Coccidiostats	Analytical Methodology	Analysed Sample	No. of Analysed Samples	Frequency (%)	Contents Min-Max (Mean) (µg/kg)	Reference
Poland	NIC, LAS, MAD, SAL, SEM, ROB	LC-MS/MS	Chicken liver	2011	2.4	8.3–2800	[65]
Poland	NIC	LC-MS/MS	Turkey liver	307	0.3	580	[65]
Poland	NIC, LAS, MAD, SAL, SEM	LC-MS/MS	Eggs	312	4.5	6.3–320	[65]
China	MAD, SAL	LC-MS/MS	Eggs	>100	<7	0.35–1.17	[68]
China	MAD	LC-MS/MS	Chicken	>100	<6	0.67–5	[68]
Canada	MON, DEC, LAS, NAR, DNC	LC-MS/MS	Chicken muscle	41	14.6	1.5–190	[69]
Japan	MAD, DIC	LC-MS/MS	Fried chicken	26	11.5	0.5–3	[70]
Japan	LAS	LC-MS/MS	Unfried chicken cutlets	20	25	0.8–2.1	[70]
Japan	NIC, DIC, SAL, MAD e LAS	LC-MS/MS	Chicken muscle	39	43.6	0.4–35.0	[70]
Greece	SAL	LC-MS/MS	Chicken tissue	29	3.4	53.5	[66]
Italy	NIC, LAS, DIC, ROB, NAR, DEC	LC-MS/MS	Chicken muscle	189	24.8	1.0–516	[30]
Italy	NIC, MAD, ROB, MON DIC, SAL, DEC	LC-MS/MS	Eggs	151	15.9	1.0–1002	[30]
Spain	NAR, MAD, MON, LAS, SAL, SEM	LC-MS/MS	milk, eggs, fat, liver, kidney, and chicken and beef muscle	14	21,4	n.d–5.6	[67]

## 10. Residue Analysis and Methodologies

Under Directive 96/23/EC, the Commission has adopted Decision 2002/657/EC, which specifies performance criteria and the interpretation of results associated with analytical methods used to monitor residues.

The presence of coccidiostats has to be monitored in animal tissues and products to ensure food safety for consumers. Table 7 groups analytical methodologies present in the scientific literature published between 2011 and 2020 related to the control of coccidiostat residues in food matrices, presenting the use of LC for the detection and quantification of these substances as a common factor, allowing the recognition of the structure of the analyte to allow its correct identification. The methodologies presented were validated as described in Commission Decision 2002/657/EC of 12 August 2002 [71].

In the study of Olejnik, M. et al. [65], the samples were analysed using a multi-residue LC–MS/MS method, validated according to the 2002/657/EC enabling the determination of 12 coccidiostats.

For the determination of six polyether antibiotics, Ref. [68] developed a method using solid phase extraction (SPE) combined with liquid chromatography-tandem mass spectrometry (LC-MS/MS). The samples were extracted with acetonitrile and purified by ENVI-Carb SPE. Thereafter, the analytes were separated on a Hypersil Gold column (2.1 × 150 mm, 5 μm) and analyzed by MS/MS detection.

Matus, J.L. et al. [69] developed a sensitive multi-residue liquid chromatography-tandem mass spectrometry (LC-MS/MS) and liquid chromatography-quadrupole time-of-flight-mass spectrometry (LC-QToF-MS). This method was validated for the determination and confirmation of residues of 17 anticoccidials, plus free ractopamine in poultry muscle. The analytes were extracted and cleaned up within a 3-hour period by simply extracting into a solvent mixture with salts followed by centrifugation, dilution, and filtration.

Buiareli, F. et al. [72] developed a sensitive, fast and robust LC-MS/MS method for the simultaneous detection of seven analytes (five compounds and two metabolites) in eggs. In this way, the possible critical factors were examined and different purification methods were tested to ascertain the best conditions and parameters for a correct and accurate extraction and purification, and for a good screening and confirmation. The composition of the mobile phase was also relevant, as it influences the peak shape and the retention behavior of the analyte in the LC column, and the best performance, in terms of mobile phase, occurred with 0.1%- formic acid in acetonitrile in gradient elution with the addition of ammonium hydroxide (0.08%). Initially, LC with UV detection was used, however, since the limits of quantification were much higher than the MRLs, the methodology was adapted to LC-MS/MS, a method capable of providing lower limits of quantification [72].

In the study by Yoshikawa, S. et al. [70], a methodology was developed capable of simultaneously determining, in processed chicken, 37 compounds, including some coccidiostats such as lasalocid, maduramicin, diclazuril and nicarbazin, through LC-MS. A correct optimization of the analytical methodology is essential when one intends to develop multi-residue methods, since the various compounds can present very different physicochemical properties. In addition to the compounds to be analysed, the fact that the matrices are complex, as in the case of processed foods (which, in addition to the food itself, may contain other substances from processing, such as fat), means that they can interfere with the analyte extraction. Thus, in this study, an extraction was performed with ethyl acetate followed by acetonitrile, since performing the extraction in two phases allowed for the extraction of analytes in lipid samples. According to the author, the methodology developed is effective, with quantification values ranging from 0.2–1.0 µg/kg and precision values ranging from 1 to 15% [70].

In the study by Barreto, F. et al. [73], 14 coccidiostats were analyzed in 619 egg samples and 2663 chicken muscle sample. Only seven egg samples and thirteen chicken muscle samples showed non-conforming results, using a multi-residue method (LC–QqLIT-MS/MS). This method proved to be fast and simple, quantitative and confirmatory. In this study, three internal standards labeled with stable isotopes (DNC-D8, DECQ-D5 and ROBE-D8) were used, to reduce the matrix effects. Since the matrices used in this study, muscle and poultry eggs, are rich in proteins and lipids, and therefore complex, it was necessary to carry out several steps of extraction and purification to obtain extracts suitable for injection into the chromatographic system. However, when cleaning is performed at low temperatures, sample preparation time is significantly reduced, simplifying the method. Therefore, in this study an extraction with can followed by cleaning at low temperatures was attempted, being an easier, faster and cheaper methodology than the conventional SPE. The described methodology allowed for good results, presenting an accuracy of 73–115% and a precision of 0.4–21%. Thus, Barreto, F. et al. [73] highlighted the importance of finding a balance between cost, speed and analytical quality [73].

Dasenaki & Thomaidis [66], developed a simple, sensitive and effective confirmation method for the determination of 16 coccidiostats in 82 samples of chicken muscle and eggs, using hydrophilic interaction liquid chromatography with tandem mass spectrometry detection (HILIC-MS/MS). Only one chicken muscle sample showed a non-conforming result. Previously, a solid–liquid extraction with acetonitrile was performed followed by purification by dispersive SPE, which allowed for cleaner extracts and better recoveries. When using HILIC, there was a greater sensitivity and better ionization efficiency, allowing for short retention times. This methodology showed high sensitivity, presenting LODs below 0.6 μg/kg for all analyzed analytes (with the lowest legislated MRLs for the matrices under study being 2 μg/kg), providing reliable and robust results [66].

Rusko et al. [74], developed a sensitive and selective multi-residue method, using liquid chromatography with detection by Orbitrap high-resolution mass spectrometry (LC-Orbitrap-HRMS), in order to determine 17 coccidiostats in birds and eggs. Extraction was performed with acetonitrile, followed by purification at moderately low temperatures (0 °C), pre-concentration, reconstitution and filtration

The study by González-Rubio, S. et al. [67] performed in 2020, in Spain, carried out the extraction using supramolecular solvents (SUPRAS) and dispersive SPE, for the determination of narasin, salinomycin, lasolacid, maturemycin, monensin and semduramycin, in samples of chicken muscle, bovine muscle and their derivatives (kidney and liver), eggs, milk and swine fat.

**Table 7:** Analytical methodologies reported in the scientific literature (2011–2020).

Sample Type	Coccidiostats	Extraction/Purification	Detection and Quantification	LOD (µg/kg)	LOQ (µg/kg)	Accuracy	Precision	Reference
chicken, turkey, eggs, water and feed	Clazuril, Diclazuril, Decoquinate, DNC, Halofuginone, Lasalocid, Maduramicin, Monensin, Narasin, Robenidine, Salinomycin, Semduramicin	Octadecyl sorbent	LC-MS/MS Column: Poroshell EC-C18, 2.7 µm, 2.1 × 150 mm. Mobile phase: (acetonitrile: methanol: 0.01 M ammonium formate at pH 4.0	10 µg/kg	0.2 µg/kg	88%	17.6%	[65]
Milk, Chicken and eggs	Lasalocid, salinomycin, monensin, narasin, madubamycin and nigericin	Acetonitrile extraction followed by alumina SPE clean-up	(SPE)-LC-MS/MS Hypersil Gold column (2.1 × 150 mm, 5 µm)	0.01–0.3 µg/kg	0.4–1 µg/kg	92–97%	-	[68]
Poultry muscle and liver, bovine muscle, liver, and kidney tissues.	Lasalocid, halofuginone, narasin, monensin, semduramicin, ethopabate, robenidine, buquinolate, toltrazuril as its sulfone metabolite, maduramicin, salinomycin, diclazuril, amprolium, decoquinate, dinitolmide, clopidol, and the nicarbazin metabolite DNC (N,N1-bis(4-nitrophenyl)urea)	Methanol (≥99.8%), acetonitrile (≥99.8%), and 2-propanol (≥99.7%)	LC-MS/MS and the LC-QToF/MS Agilent Poroshell 120 EC-C18 analytical column (2.1 × 100 mm, 2.7 µm) held at 55 °C with a 3 μL injection volume of all samples and standards	0.2–91 µg/kg	0.6–305 µg/kg	49–104%	0.3 to 10%	[69]
Eggs	Clazuril, DIC, ROB, NIC, toltrazuril (and its 2 metabolites)	Solid-liquid extraction and purification (SPE)	LC-MS/MS Column Phenomenex C_18_ (5 µm, 150 mm × 2.1 mm) Mobile phase: 0.1% aqueous formic acid (A) and Ammonium hydroxide (0.08%) (B)	CCα: 2.2−320.0 µg/kg	CCβ: 2.6−350.0 µg/kg	80% (62% was minimum for robenidine and 95% was maximum for toltrazuril)	2.9−14.7% Repeatability: 4.1–13.0% Intra-laboratory reproducibility: 6.4–14.1%	[72]
Fried chicken, non-fried chicken cutlet and chicken muscle	37 compounds belong, including LAS, MAS, MON, NAR, SAL, SEM, DEC, DIC, NIC	Acid extraction (SLE with ethyl acetate and acetonitrile)	LC-MS/MS Column: InertSustainSwift C_18_ (2.1 mm i.d. × 150 mm, 5 µm; GL Sciences) Mobile phase: 0.1% formic acid in 10 mmol/L ammonium acetate (A) and methanol (B)	Not applicable	0.2–1.0 µg/kg	70–105%	Repeatability: 1–11% Intra-laboratory reproducibility: 1–15%	[70]
Broiler muscle and eggs	LAS, MAD, MON, NAR, SAL, SEM, ROB, DIC, toltrazuril, trimethoprim, clopidol, amprolium, diaveridine and NIC	Acetonityl extraction	LC–QqLIT-MS/MS Column: Poroshell 120 EC-C18 coupled to a C18 column Mobile phase: gradient of water and acetonitrile	10% of MRL	25% of MRL	73–115%	0.4–21%	[73]
Animal muscle (chicken, pork, beef, rabbit) and eggs	LAS, amprolium, MON, NAR, MAD, ROB, DEC, NIC, clopidol, HAL, etopabate, diaveridine, arprinocide, DIC, SEM and nigericin.	Solid-liquid extraction with acetonitrile and dispersive SPE	HILIC-MS/MS Column: ACQUITY UPLC BEH HILIC Mobile phase: acetonitrile (A) and ammonium formate with formic acid (B)	0.004–0.560	0.004–0.560	79.1–118%	Muscle: 5.3–20% Eggs: 6.4–17%	[66]
Chicken, pork, beef and fish muscles; chicken eggs; cow milk; pork offal.	20 compounds, including buquinolate, clopidol, closantel, DEC, diaveridine, DIC, dimetridazole, etopabate, HAL, imidocarb, isometamide, levamisole, metronidazole, NIC	acetonitrile/methanol containing formic acid, sodium cetate and magnesium sulfate; purification with n-hexane saturated with acetonitrile	LC-MS/MS Column: Agilent Poroshell 120SB C18 (2.7 mm, 3.0 mm × 150 mm) Mobile phase: methanol (with 0.1% formic acid) and 5 mM ammonium formate	Not applicable	2–5 µg/kg	Broiler muscle recovery: 75.1–118.9%	Chicken muscle: Intra day accuracy: 1.7–40.5% Interday accuracy: 3.4–43.3%)	[75]
Broiler muscle and eggs	Amprolium, clopidol, DEC, MON, nequinato, toltrazuril, toltrazuril sulfona, e toltrazuril, sulfoxide, DIC, LAS, SAL, HAL, MAD, NAR, NIC, ROB e SEM	Extraction with 20 mL of acetonitrile	LC-HRMS Column: Kinetex C_18_ Mobile phase: water, acetonitrile and methanol	Eggs: CCα: 2.2–336 µg/kg Chicken muscle: CCα: 2.64–589 µg/kg	Eggs: CCβ: 2.58–401 µg/kg Chicken muscle: CCβ: 3.74–749 µg/kg	Eggs: 94.1–105.8% Chicken muscle: 91.6–105.7%	Eggs: 5.2–21.3% Chicken Muscle: 5.2–20.4%	[74]
Chicken muscle, beef muscle and its derivatives: kidney and liver, eggs, milk and pork fat.	Narasin, salinomycin, lasalocid, maturemycin, monensin and semduramycin.	Extraction with supramolecular solvents and dispersive SPE.	LC-MS/MS Column: Phenomenex Luna C18 Injection volume: 2 µL Mobile phase: formic acid (0.1%) in water (A) and formic acid (0.1%) in methanol (B) Flow: 250 µL/min	0.004–0.07	-	71–112%	14 samples of which 3 samples are non-conforming (pig fat, beef kidney and eggs)	[67]

## 11. Responsibilities of Food Business Operators

Food business operators have to assure that food products observe the veterinary medicine residue and contaminant limits allowed under legislation. Veterinarians and livestock producers are responsible for drug withdrawal periods, and for ensuring that residues do not accumulate in foods. Veterinarians must abstain from the use of unapproved or illegal drugs to help control medical residues [76].

## 12. Concluding Remarks

A public health risk may arise from the presence of unauthorised substances, residues of veterinary drugs or chemical contaminants in food.

Coccidiosis continues to be one of the major disease problems of the chicken industry. In the EU, (prophylactic) coccidiostats or anticoccidial drugs remain necessary for modern animal husbandry.

Although the last EFSA report (2017) shows a very low rate of excessive residues due to anticoccidials, and a decrease since 2009, the use of coccidiostats must be under veterinary prescription, as this would allow the choice of a first-rate approach to eliminate the use of coccidiostats in the long term, and to extend the useful life of coccidiostats by minimising resistance, as well as to report adverse reactions, including lack of efficacy and, above all, to ensure that withdrawl periods are honoured.

Based on Regulation (EC) No 1831/2003, the current system has proven effective and is well prepared to deal with the current situation. It provides a high level of safety for consumers, adequately protects the health and welfare of animals, as well as of the environment, and provides an environment in which economic operators can operate in a fair manner.

On the other hand, the Federation of European Veterinarians (FVE) states that coccidiostats should be considered as antibiotics, and a prescription for their use should be mandatory.

Based on scientific data, it is imperative to eliminate coccidiostats in the future and to make changes in management today, to prevent chronic toxicity caused by long-term exposure to low levels of coccidiostats and to preserve the efficacy of the currently available anticoccidials.

In summary, poultry production would not have evolved into the highly efficient meat production industry that it is without the help of ionophores for the prevention of coccidiosis. The removal of this crucial element of the coccidiosis control toolbox would unavoidably cause a reduction in poultry production performance, resulting in lower outputs and jeopardizing animal health and welfare. Due to the nature of poultry production and the features of coccidiosis, the prevention of coccidiosis is crucial for remaining competitive and ensuring animal welfare and health. Prevention can only be achieved using all the available tools, which include chemical products, vaccines and ionophores in rotation programs. Using these tools at different time points is the most efficient and viable long-term strategy.

## Figures and Tables

**Table 1:** *Eimeria* species and respective.

Species	Lifecycle-Duration
*Eimeria acervulina*	5 days
*Eimeria brunetti*	6 days
*Eimeria maxima*	7 days
*Eimeria mivati*	5 days
*Eimeria necatrix*	7 days
*Eimeria praecox*	4 days
*Eimeria tenella*	7 days

**Table 2:** Maximum residue limits and withdrawal period for each compound, species and edible tissues.

Generic Name/Applicable Legislation	Target Species	LMR	Withdrawal Period
**Monensin** (Comission Regulation No. 1096/2008)	Broiler	25 μg/kg of skin/ fat; 8 μg/kg of liver, kidney and muscle.	1 day
	Turkeys (max. 16 weeks)		1 day
	Laying hens	2 μg/kg of skin /fat, kidney and muscle; 8 μg/kg of liver.	1 day
**Salinomicin** (Comission Regulation No. 496/2007)	Broiler	150 µg/kg of liver; 40 µg/kg of kidney; 15 µg/kg of muscle, 150 µg/kg of skin/fat.	0 day
	Laying hens	5 µg/kg of liver; 2 µg/kg of kidney; 2 µg/kg of muscle, 2 µg/kg of skin/fat.	0 day
	Eggs	3 μg/kg	-
**Narasin** (Comission Regulation No. 885/2010)	Broiler	50 µg/kg all edible tissues	1 day
	Eggs	2 μg/kg	-
**Lasalocid** (Comission Regulation No. 37/2010)	Broiler	20 μg/kg of muscle; 100 μg/kg of skin/fat, liver; 50 μg/kg of kidney	5 days
	Turkey		5 days
	Laying hens	-	5 days
	Pheasants, guinea fowl, quails and partridges, other than poultry.	-	5 days
	Eggs	5 μg/kg	-
**Maduramicin** (Comission Regulation No. 388/2011)	Broiler	150 μg/kg of liver, skin/fat; 100 μg/kg of kidney; 30 μg/kg of muscle	3 days
	Turkey (max. 16 weeks)	-	5 days
	Eggs	12 μg/kg	-
**Robenidine** (Commission Regulation No. 124/2009 and Community register of feed additives)	Broiler	800 μg/kg of liver; 350 μg/kg of kidney; 200 μg/kg of muscle; 1 300 μg/kg of skin/fat	5 days
	Turkeys	400 μg/kg of skin/fat 400 μg/kg of liver 200 μg kg of kidney 200 μg/kg of muscle	5 days
	Eggs	25 μg/kg	-
**Halofuginone** (Commission Regulation No. 37/2010 and Commission Regulation No. 124/2009)	Broiler	-	5 days
	Turkeys (max. 12 weeks)	-	5 days
	Eggs	6 μg/kg	-
**Diclazuril** (Comission Regulation No. 971 and 976/2008)	Broiler	1500 μg/kg of liver; 1000 μg/kg of kidney; 500 μg/kg of muscle; 500 μg/kg of skin/fat.	-
	Turkeys		-
	Laying hens	-	-
	Eggs	2 μg/kg	-
**Decoquinate** (Commission Regulation No. 37/2010)	Broiler	1000 μg/kg liver and skin/fat; 800 μg/kg of kidney; 500 μg/kg of muscle	-
**Nicarbazin** (Commission Regulation No. 124/2009)	Broiler	15,000 μg de DNC/kg of liver; 6000 μg de DNC/kg of kidney; 4000 μg de DNC/kg of muscle and skin/fat	1 day
	Eggs	300 μg/kg	-

**Table 3:** Production of poultry and number of target samples over 2007–2019 [63,64].

Year	Production (t)	Targeted Samples	% Samples Tested/200 t (a)	Minimum 96/23/EC
2007 (EU 27)	10,912,500	62,101	1.15	1/200 t
2008 (EU 27)	12,421,566	60,406	1.11	
2009 (EU 27)	11,383,434	61,989	1.00	
2010 (EU 27)	11,804,262	61,259	1.08	
2011 (EU 27)	12,417,108	65,942	1.12	
2012 (EU 27)	12,845,333	68,770	1.11	
2013 (EU 28)	12,930,555	71,186	1.11	
2014 (EU 28)	12,909,837	72,486	1.12	
2015 (EU 28)	13,394,013	71,223	1.10	
2016 (MS 27 (b))	12,239,495	64,501	1.10	
2016 (EU 28)	13,906,572			
2017 (EU 28)	14,320,889	67,630	0.97 (c)	
2018 (EU 28)	14,683,847	69,096	0.96	
2018 (EU 27 (d))	14,789,918			
2019 (EU 27 (d))	15,186,857	73,088	0.99	

(a) in relation to the production of the previous year; (b) data from France were not available for inclusion in the 2016 results report; (c) calculated based on 2016 production data from 28 Member States (MS); (d) The 2019 results data from Malta were not available for inclusion in this report.

**Table 4:** Production volume and number of targeted samples collected for poultry [63]. According to Directive 96/23/EC, the minimum number of samples for each category of poultry must be one per 200 t of annual production.

Country	Production Data (t) (a)	Number of Samples 2019	Samples Tested/200 t
Austria	127,714	824	1.29
Belgium	396,757	2132	1.07
Bulgaria	110,767	480	0.87
Croatia	56,669	372	1.31
Cyprus	27,151	261	1.92
Czechia	159,076	951	1.2
Denmark	152,419	765	1.0
Estonia	19,434	200	2.06
Finland	128,446	634	0.99
France	1,705,840	6583	0.77
Germany	1,567,973	9530	1.22
Greece	243,193	630	0.52
Hungary	675,965	3240	0.96
Iceland	9484	208	4.39
Ireland	180,843	1413	1.56
Italy	1,354,000	6913	1.02
Latvia	34,000	182	1.07
Lithuania	89,256	446	1
Luxembourg	0	NA	NA
Netherlands	968,373	4941	1.02
Norway	100,263	655	1.31
Poland	2,173,741	8721	0.8
Portugal	353,227	2148	1.22
Romania	475,952	2540	1.07
Slovakia	104,686	661	1.26
Slovenia	61,414	322	1.05
Spain	1,528,845	7170	0.94
Sweden	158,430	826	1.04
United Kingdom	1,826,000	9340	1.02
Total	14,789,918	73,088	0.99

(a) in relation to the production of the previous year.

**Table 5:** Residues in live animals and animal products—Results 2019 [63]. Note: Under Directive 96/23/EC, Group B substances comprise approved veterinary medicines, such as antimicrobials, antiparasitics, sedatives, anticoccidials.

Group	Substance	Member State	Number of Samples Analysed	Non-Compliant Results	% Non-Compliant Results
B2b	Halofuginone	Italy	357	1	0.28
B2b	Lasalocid	France	740	1	0.14
B2b	Monensin	The United Kingdom	1493	1	0.07
B2b	Toltrazurilsulfon	The Netherlands	299	1	0.33
B2b	Sub-total for B2b	4	NA	4	NA

## Data Availability

No new data were created or analyzed in this study. Data sharing is not applicable to this article.
